# A miniature multi-contrast microscope for functional imaging in freely behaving animals

**DOI:** 10.1038/s41467-018-07926-z

**Published:** 2019-01-09

**Authors:** Janaka Senarathna, Hang Yu, Callie Deng, Alice L. Zou, John B. Issa, Darian H. Hadjiabadi, Stacy Gil, Qihong Wang, Betty M. Tyler, Nitish V. Thakor, Arvind P. Pathak

**Affiliations:** 1 Department of Biomedical Engineering, Johns Hopkins University School of Medicine, Baltimore, MD 21205 USA; 2 Russell H. Morgan Department of Radiology and Radiological Science, Johns Hopkins University School of Medicine, Baltimore, MD 21205 USA; 3 Department of Neurosurgery, Johns Hopkins University School of Medicine, Baltimore, MD 21205 USA

## Abstract

Neurovascular coupling, cerebrovascular remodeling and hemodynamic changes are critical to brain function, and dysregulated in neuropathologies such as brain tumors. Interrogating these phenomena in freely behaving animals requires a portable microscope with multiple optical contrast mechanisms. Therefore, we developed a miniaturized microscope with: a fluorescence (FL) channel for imaging neural activity (e.g., GCaMP) or fluorescent cancer cells (e.g., 9L-GFP); an intrinsic optical signal (IOS) channel for imaging hemoglobin absorption (i.e., cerebral blood volume); and a laser speckle contrast (LSC) channel for imaging perfusion (i.e., cerebral blood flow). Following extensive validation, we demonstrate the microscope’s capabilities via experiments in unanesthetized murine brains that include: (i) multi-contrast imaging of neurovascular changes following auditory stimulation; (ii) wide-area tonotopic mapping; (iii) EEG-synchronized imaging during anesthesia recovery; and (iv) microvascular connectivity mapping over the life-cycle of a brain tumor. This affordable, flexible, plug-and-play microscope heralds a new era in functional imaging of freely behaving animals.

## Abstract (web summary)

Measuring multiple neurophysiologic variables usually requires bulky benchtop optical systems and working with anesthetized animals. Here the authors present a miniature portable microscope for neurovascular imaging in awake rodents, combining fluorescence, intrinsic optical signals and laser speckle contrast.

## Introduction

Neurovascular coupling constitutes the biophysical basis of functional imaging methods such as functional magnetic resonance imaging (fMRI)^1^, and resting-state fMRI (rs-fMRI)^2^. Moreover, neuropathologies ranging from Alzheimer’s disease^3^ to brain tumors^4^ involve a dysfunctional neurovascular unit and cerebrovascular remodeling. Therefore, there is a crucial need for new tools that can interrogate these phenomena in the unanesthetized mammalian brain. Characterizing neurovascular coupling in the brains of awake and freely behaving animals requires the ability to image neuronal firing and concomitant changes in cerebral hemodynamics. Analogously, characterizing neuropathology-induced alterations requires the ability to track fluorescently tagged cells along with microvascular remodeling. Although advances in optical engineering have enabled the development of miniaturized microscopes for in vivo neuroimaging in freely behaving or unanesthetized animals^5^, most current systems are limited to performing either neural^6–8^ (i.e., fluorescence) or hemodynamic imaging^9–11^ (i.e., intrinsic optical signal or laser speckle contrast), but not both.

Multi-contrast optical neuroimaging systems^12,13^ enable us to simultaneously interrogate multiple neurophysiologic variables (e.g., neuronal and hemodynamic responses), a feat not possible with traditional single-contrast optical imaging techniques^14–16^. This approach has enabled neuroscientists to visualize the multiple facets of brain function^2,17,18^ and dysfunction^19,20^ in real-time, at optical spatial resolutions. However, imaging systems that combine multiple optical contrast mechanisms tend to be expensive and bulky because of the substantial footprint of their benchtop-based electronic and optical components. Moreover, such multi-contrast imaging systems are often custom fabricated and not commercially available. This makes them unsuitable for mass production or widespread adoption by neuroscientists.

In addition, their bulk reduces portability and often requires the animal to be anesthetized, which can alter the physiology being imaged. Currently this hurdle is circumvented by conducting imaging on head-fixed animals^21–23^. However, head fixation is unsuitable for experiments in which correlation with unrestrained animal behavior is required^24,25^. Furthermore, use of head-fixation in many experimental paradigms necessitates additional customized designs of complex virtual reality environments to permit realistic simulation of surroundings for the rodent^26,27^. Finally, the space constraints of conventional benchtop imaging systems can preclude the use of complementary recording equipment, such as EEG arrays.

To circumvent the abovementioned issues, we developed a miniaturized microscope capable of in vivo neurovascular imaging in freely moving or awake animals. We achieved this by integrating three optical contrast mechanisms into our microscope: fluorescence (FL)^6^, intrinsic optical signals (IOS)^28^, and laser speckle contrast (LSC)^29^. The FL channel enables imaging of neural activity with voltage sensitive dyes^30^ or via genetically encoded calcium indicators such as GCaMP^31^. The FL channel can also be used for tracking the fate of fluorescently tagged cells (e.g., tumor cells and gene reporter systems). The IOS channel enables imaging of the total hemoglobin (HbT) absorption, which is proportional to the cerebral blood volume (CBV) and also permits imaging of microvascular morphology, while the LSC channel enables imaging of the cerebral blood flow (CBF). This multi-contrast microscope costs significantly less than similarly equipped benchtop imaging systems due to our use of commercially available miniaturized components such as LEDs, a micro-lens, a CMOS image sensor, as well as an entirely 3D printed microscope housing. We also included a synchronization (sync) channel in this microscope. This unique feature permits image acquisition to be time-locked with complementary recording systems (e.g., EEG), or systems that provide a stimulus to the animal (e.g., an aural tone generator). Our fully portable plug-and-play microscope weighs 3 g and occupies 5 cm^3^, and is well suited for in vivo neuroimaging in freely behaving mice and rats. The microscope has a spatial resolution of 5 μm, which enables individual microvessels to be resolved. It covers a 3 × 3 mm^2^ wide-field (or mesoscopic) field of view (FoV) that permits the interrogation of entire cortical regions (e.g., the auditory cortex). The microscope can acquire images at a maximum rate of 15 frames per second (fps), permitting dynamic neurovascular events to be captured with high fidelity.

We first present the design of the multi-contrast miniature microscope followed by extensive validation of its performance against a similarly equipped benchtop-based imaging system. Next, we demonstrate its utility in awake or freely behaving animals by performing proof-of-principle experiments that exploit its multi-contrast capabilities to interrogate in vivo neural activity, image concomitant hemodynamic changes, explore correlations with EEG, and track angiogenesis-induced changes in cerebrovascular structure and function over the entire life-cycle of a brain tumor.

## Results

### Miniature plug-and-play design with three optical contrast mechanisms

In contrast to previously reported high-magnification/small field of view (FoV) miniaturized microscope designs^6,7,22^, ours trades magnification in favor of a larger (3 × 3 mm^2^) FoV to ensure greater cortical coverage. Moreover, the resulting large object distance enables us to accommodate the multiple illumination sources necessary for multi-contrast imaging, while allowing us to maintain a spatial resolution of 5 μm.

**Fig. 1:**
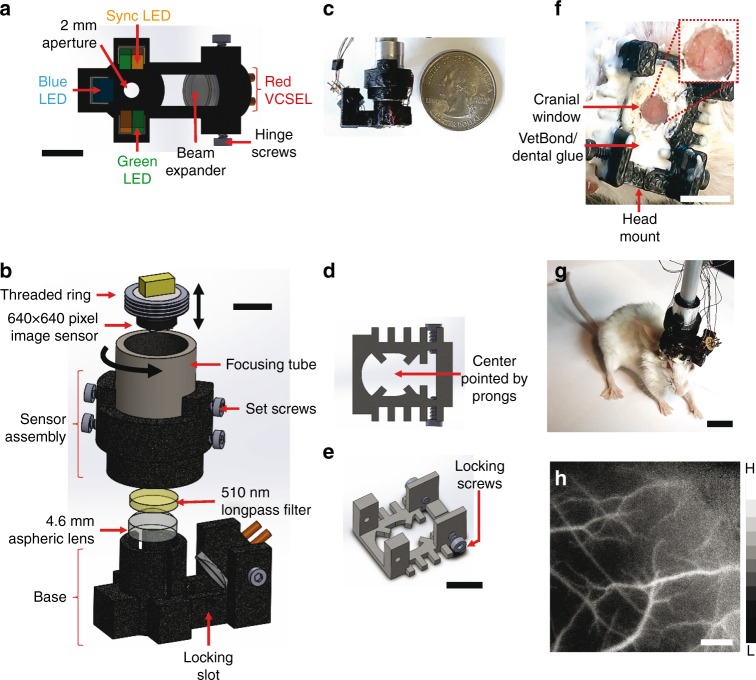
Miniature plug-and-play design with three optical contrast mechanisms. **a** A bottom view of the microscope base unit. Excitation for green fluorescent imaging is provided by a blue LED, while HbT imaging is carried out with a pair of green LEDs. A red laser diode in conjunction with a beam expander and hinge screws facilitates dHb and LSC imaging. A separate pair of orange (sync) LEDs is used for synchronizing with external instruments (e.g., an EEG system). The FoV lies directly below the aperture. **b** The sensor unit of the microscope shown along with the base consists of a 4.6 mm focal length lens, a 510 nm long-pass filter to block blue fluorescence excitation light, a focusing tube and an image sensor. **c** The complete microscope assembly is shown with a U.S. quarter coin next to it for scale. **d**, **e** Bottom and side views of the head mount, respectively. The head mount is surgically implanted on the rodent’s skull and enables firm attachment of the microscope via a pair of locking screws. The pronged structure at the base of the head mount facilitates centering on the FoV. **f** Head mount attached to a mouse skull with the inset showing the cranial window for optical access to the brain. **g** A freely moving mouse with the microscope. **h** A grayscale CBF map acquired with the microscope from an awake mouse using LSC imaging. The microscope housing was rapid prototyped and 3D printed, which permitted a high degree of customization. Scale bar indicates 5 mm in **a**, **b**, and **f**, 1 cm in **g** and 500 µm in **h**. Also see Supplementary Figs. 1, 2 and 3

The microscope comprises a 3D printed base and sensor assembly (Fig. 1a, b). The base houses the three illumination sources (Fig. 1a): a blue (452 nm) LED provides excitation for fluorescence imaging; green (570 nm) LEDs and a red (680 nm) laser diode provide illumination for IOS imaging of total hemoglobin (HbT, at 570 nm) and deoxyhemoglobin absorption (dHb, at 680 nm), respectively. Additionally, speckle contrast is computed from images acquired under red laser diode illumination, enabling us to map in vivo cerebral blood flow (CBF). A pair of orange LEDs provides an optical sync  signal for synchronizing image acquisition with external instruments or recording hardware (e.g., EEG). The base also contains the elements necessary for light filtering and image formation. The 510 nm long-pass filter rejects blue excitation light (<510 nm) while permitting light from the other illumination sources to reach the sensor. A single (*f* = 4.6 mm) aspheric lens was used for image formation (Fig. 1b).

The 3D printed sensor assembly contains the elements necessary for focusing and sensing (Fig. 1b). Images were acquired using a 640 × 640 pixel, 10-bit CMOS image sensor (NanEYE GS, Awaiba Holdings SA, Switzerland) with a pitch of 3.6 µm. The complete miniature microscope (Fig. 1c) assembly weighed 9 g, occupied 5 cm^3^, was directly powered and controlled by a laptop computer, and can acquire images at 15 fps. Finally, we designed a strain relief mechanism that reduced the head-borne weight of the entire microscope assembly to 3 g (Supplementary Fig. 1a). We assayed blood corticosterone levels and demonstrated a gradual reduction in stress to the animal following habituation with the head mount and microscope (Supplementary Fig. 1b). Simultaneously acquired videos of animal behavior following habituation confirmed that the head-borne microscope did not impede free movement or natural behavior of the animals during experiments (Supplementary Movie 1).

### A disposable head mount enables reuse of the microscope

We designed a customized, disposable head mount (Fig. 1d,  e; see Methods) for the microscope to interface with the rodent brain. Each head mount was 3D printed and attached permanently over a surgically prepared cranial window (Fig. 1f, see Methods). Set screws within the head mount enable robust attachment and minimized microscope motion (Fig. 1g) during high-resolution in vivo imaging (Fig. 1h). Loosening the set screws permits the microscope to be conveniently detached and reattached whenever the experimental protocol demands it. Customized head mounts can be 3D printed to match the type of rodent being imaged.

### Miniaturized control modules and GUI enhance plug-and-play portability

We supplemented the microscope system with two compact control modules: an illumination controller (see Methods and Supplementary Fig. 2) and a commercially available image acquisition controller (see Methods). Collectively, they miniaturized the hardware needed for illumination current generation and high-speed image acquisition. A flexible wire bundle linked the head-mounted microscope to the control modules, which were powered by USB connections to a standard laptop computer (see Methods and Supplementary Fig. 3a, b). This configuration resulted in a portable imaging system that could be transported to any experimental site for imaging in awake or freely behaving animals (Supplementary Fig. 3c). We also custom designed an all-in-one graphical user interface or GUI (Supplementary Fig. 3d) to control image acquisition parameters and provide real-time image visualization. Collectively, these design features enhanced the plug-and-play utility of the portable imaging system.

### Microscope performance was comparable to a similar benchtop imaging system

**Fig. 2:**
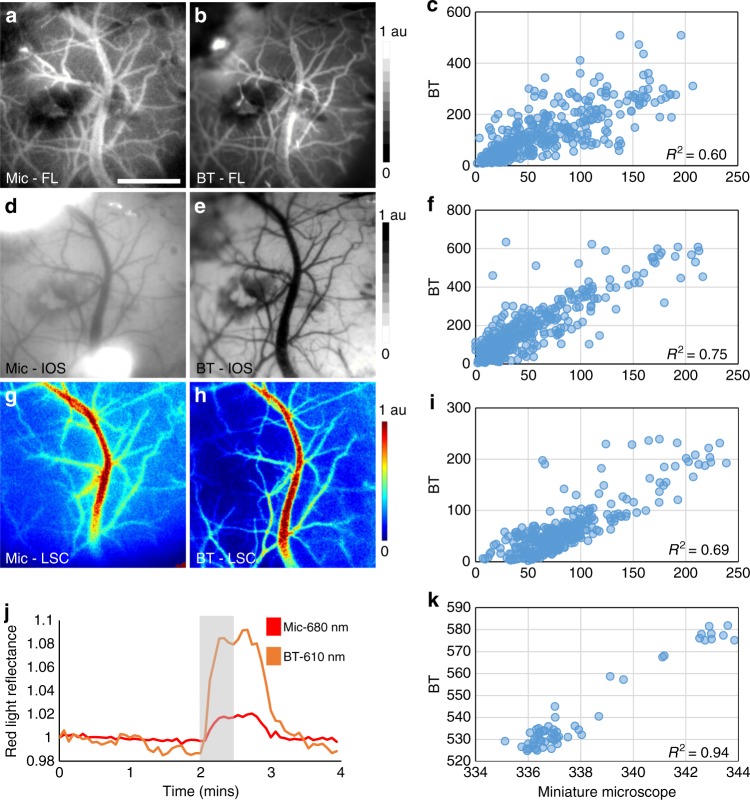
Performance was comparable to a similar benchtop imaging system. Multi-contrast images of a mouse brain acquired using our microscope (Mic) and a similarly equipped benchtop (BT) imaging system were compared. **a**, **b** A network of cerebral microvessels visualized with fluorescence imaging (FL) following a tail vein injection of dextran-FITC. **d**, **e** The same FoV imaged under green light illumination (IOS) illustrating HbT (or CBV) distribution. **g**, **h** Pseudo-colored maps of CBF obtained by performing laser speckle contrast imaging under laser illumination (LSC). **j** Time courses of the dHb-dependent red light reflectance from a central 20×20 pixel FoV showing the temporal response to an oxygen inhalation challenge (i.e., inhalation of 100% O_2_ for 30 s starting at 2 mins, followed by room air for the remainder of the experiment). **c**, **f**, **i**, **k** Scatter plots of the post-processed  in vivo BT vs. Mic measurements shown in **a**–**h,** axes are signal intensity in arbitrary units (a.u.). Spatial correlations were computed for a 20 × 20 pixel grid superimposed over a central 400×400 region. Data presented is from a single animal and scale bar indicates 1 mm

To validate microscope performance for each optical contrast mechanism, we performed wide-field micro-angiography of an anesthetized mouse brain and imaged the same FoV with a similarly equipped multi-contrast benchtop system (see Methods). Images of cortical microvasculature obtained from the two systems with fluorescence (Fig. 2a, b), IOS (Fig. 2d, e) and laser speckle contrast (Fig. 2g, h) were compared for a central 400 × 400 pixel region of interest (ROI) overlaid with a 20×20 pixel sampling grid for analysis. For each contrast mechanism, correlation coefficients (*R*^2^) of 0.60, 0.75, and 0.69 were observed between the benchtop and microscope derived imaging data, respectively (Fig. 2c, f, i). As additional validation, red light reflectance was used to track temporal changes in dHb levels following an oxygen inhalation challenge. Time series for each imaging system from a central 20 × 20 pixel region exhibited an increase in the red reflectance signal (Fig. 2j), i.e., a decrease in dHb, as well as an *R*^2^ of 0.94 (Fig. 2k). Collectively, these data validate the ability of our microscope to conduct in vivo imaging using each optical contrast mechanism.

### Localized neural and global hemodynamic response to auditory stimulation

**Fig. 3:**
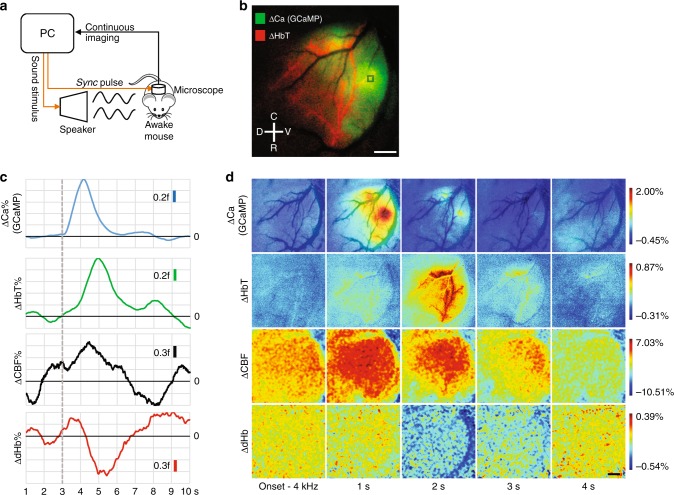
Localized neural and global hemodynamic response to auditory stimulation. **a** Schematic of the experimental setup for the auditory stimulation and image acquisition. **b** A composite (i.e., RGB) map showing the maximum calcium response (green channel) and the corresponding maximum vasodilatory response (HbT, red channel) to a 4 kHz stimulus. Anatomical directions dorsal (D)—ventral (V), caudal (C)—rostral (R) are provided for reference. The black background image is a vessel mask (i.e., binarized version of the blood vessel image) underlay. **c** Time series of the calcium and hemodynamic responses to a 4 kHz stimulus from the 20 × 20 pixel region of interest (ROI) shown in **b**. The ordinates for each plot are in fractions (**f**) of the peak response amplitude. The black line indicates the baseline for each trace and the hashed gray line indicates when the stimulus was presented. A sliding 1 s window was used to smooth the time traces. **d** Snapshots illustrating the spatiotemporal evolution of the neurovascular response to 4 kHz stimulation. Each image has been smoothed using a 3 × 3 pixel median filter and normalized to 0.1% of its range. For visual clarity, the CBF and dHb images were additionally smoothed with a 20 × 20 mean filter before normalization. A vessel mask underlay is used in the first row of images to provide a visual reference for the microvasculature. Data presented is an average over 30 trials from a single animal and scale bar indicates 500 μm. *Also see* Supplementary Fig. 4 and Supplementary Movies 2-5

We performed multi-contrast imaging of the mouse auditory cortex in response to sound stimuli in an awake mouse (Fig. 3a, see Methods). Here, we present results following a 4 kHz auditory stimulus. We successfully captured the time evolution of neural activation (via GCaMP bound calcium fluorescence^32^) and the concomitant hemodynamic response in terms of changes in CBV (i.e., total hemoglobin concentration or HbT), CBF and oxygenation (i.e., dHb) (Fig. 3b and Supplementary Movies 2–5). Time-series analyses revealed the classic hyperemic lag between neural firing and the onset of the hemodynamic response^13^ (Fig. 3c, see Methods). The sharp rise in calcium accumulation due to neural firing was followed by increases in total hemoglobin (HbT) and CBF, and a decrease in deoxyhemoglobin (dHb) with a delay of ~1 s. The calcium activity showed strong spatial specificity to distinct regions of the auditory cortex (Fig. 3d, ΔCa%). Vasodilation, as indicated by the increase in HbT, exhibited the preferential recruitment of a specific microvascular irrigation territory to cater to this increased metabolic demand (Fig. 3d, ΔHbT%). In contrast, elevated CBF levels were observed globally and did not show any spatial specificity (Fig. 3d, ΔCBF%). Moreover, most of the auditory cortex exhibited an increase in HbT along with a decrease in dHb (Fig. 3d, ΔdHb%), suggesting that much of the post-stimulus perfusion to the region was hyper-oxygenated. Finally, by comparing the pre-stimulus patterns in both Fig. 3c, d, we observed that the stimulus-evoked CBF response rode on top of a large non-stimulus (or resting-state) related fluctuation that was spatially non-specific. While further studies are needed to derive mechanistic conclusions about neurovascular coupling, these data demonstrate the utility of our multi-contrast microscope in characterizing the dynamics of the neurovascular response over an entire cortical region in response to a stimulus.

### Wide-area functional mapping analogous to stimulus-based fMRI

**Fig. 4:**
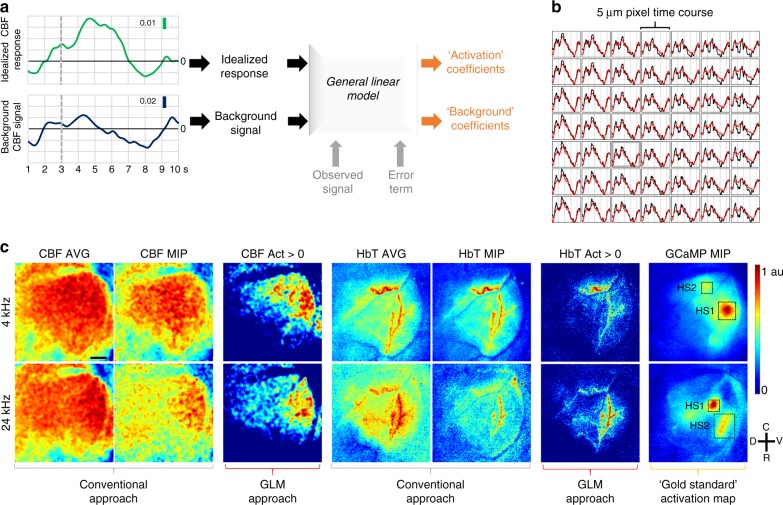
Wide-area functional mapping analogous to stimulus-based fMRI. **a** Schematic of the image processing pipeline. The idealized response and the background signal were used as regressors to compute the activation and background coefficients from observed time courses using a general linear model (GLM). **b** AFNI screenshot showing pixel-wise CBF time courses and their corresponding model fits (black: observed CBF response, red: model fit) over a 7 × 7 pixel area. **c** CBF and HbT average (3–7s), maximum intensity projected (3– 7 s) (MIP) and positive activation coefficient (Act) maps for 4 and 24 kHz stimuli. GCaMP MIPs were employed as the gold standard. All maps were normalized to 0.1%. Two GCaMP hot-spots (HS1 and HS2) are marked on each GCaMP map. One can see that the positive CBF and HbT activation coefficient maps exhibit more tonotopic specificity to the GCaMP signal (i.e., neural activity) than the average or maximum intensity projected CBF and HbT maps. Data presented is an average over 30 trials from a single animal and scale bar indicates 500 μm

Stimulus-based functional magnetic resonance imaging (fMRI) techniques^33^ have been used extensively over the past three decades to map neuronal activity in different cortical regions^34,35^. Typically, the stimulus-evoked blood-oxygen-level-dependent (BOLD) fMRI signal is treated as a surrogate of neuronal activation and used as an input to a pixel-wise General Linear Model (GLM) to identify activated pixels (see Methods). This process involves modeling the BOLD response observed in each pixel as a combination of an idealized response and background signal (Fig. 4a). The GLM approach is  in  contrast to  conventional methods of identifying activated pixels in optical imaging experiments wherein one usually calculates an average or maximum intensity projected (MIP) signal over a desired FoV without computing the pixel-wise response. While several groups have reported GLM based approaches for analyzing optical imaging data, these have been mostly limited to a single-contrast mechanism^36,37^ (e.g., hemoglobin absorption in the near infrared spectrum). Here we used the Analysis of Functional Neuro Images (AFNI)^38^ fMRI processing software to generate GLM-based wide-area activation maps of auditory cortical tonotopy by exploiting the multi-contrast capabilities of the microscope.

For our multi-contrast GLM approach, the map of the neuronal response (i.e., GCaMP fluorescence) to the 4 kHz stimulus served as the gold standard for identifying activated brain areas. We first computed an idealized CBF response (i.e., an activation CBF time-course) by subtracting the background CBF signal from the CBF signal corresponding to the GCaMP hot-spot (see Supplementary Fig. 4 and Methods). We then used the idealized CBF response and the background CBF signal as inputs to a GLM that computed fits for the observed CBF time course in each pixel (Fig. 4a–b and see Methods). The resulting activation coefficients (see Methods) were mapped on a pixel-wise basis for the entire auditory cortex analogous to classic fMRI paradigms. The conventional average and maximum intensity projected CBF signals for each pixel were also computed (Fig. 4c). To determine if we could map the tonotopy of the auditory cortex, we applied the same GLM pipeline to CBF responses following a 24 kHz stimulus. In addition to the CBF response, we repeated the GLM approach for the HbT response, but not the dHb response because of its low signal-to-noise ratio (SNR). The neuronal response to 4 and 24 kHz tones as assessed from the GCaMP maps, exhibited distinct spatial localization (i.e., tonotopy) in accordance with previous studies of the murine auditory cortex^21^, (Fig. 4c, GCaMP MIP). Following the GLM analysis, the positive CBF and HbT activation coefficient maps (Fig. 4c, CBF, HbT Act maps) exhibited greater tonotopic specificity (relative to the Ca response) corresponding to the 4 and 24 kHz stimuli than either the average or MIP CBF and HbT maps (Fig. 4c, CBF, HbT AVG, and MIPs). These proof-of-principle results showcase the power of combining an fMRI type wide-area analytical framework with multi-contrast datasets readily acquired by a miniature microscope such as ours.

### Synchronized multi-contrast imaging and EEG during anesthesia recovery

**Fig. 5:**
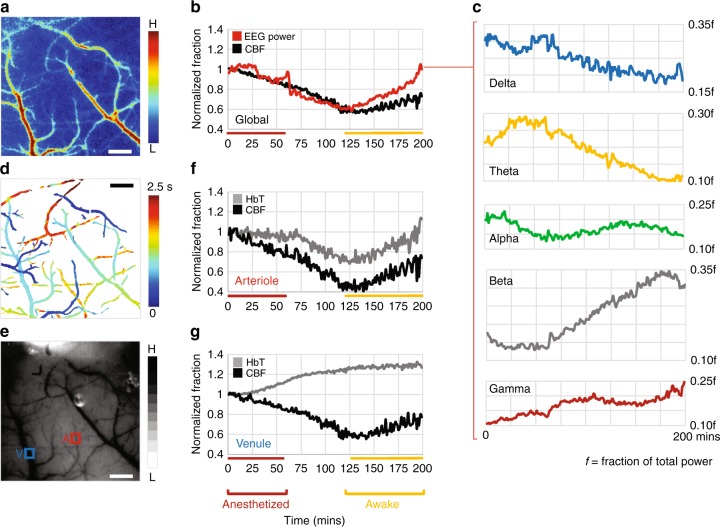
Synchronized multi-contrast imaging and EEG during anesthesia recovery. **a** Map of the average CBF over the duration of the experiment. **b** Fluctuations in global EEG power and CBF are correlated. The anesthetized and awake phases are indicated by colored bars. **c** Plots of the power in EEG sub-bands showing how each varied during recovery from anesthesia. The post-script ‘*f*’ depicts the power of each sub-band as a fraction of the total EEG power. **d** Pseudocolored map illustrating the differences in arrival time of an intravenously injected fluorescent tracer (dextran-FITC). Arrival times of the fluorescent tracer are scaled for visualization purposes within the FoV. **e** IOS map acquired under green light illumination showing HbT absorption for the same FoV. An arteriole (A) and venule (V) are identified in the FoV. **f**, **g** Time-series illustrating HbT (i.e., CBV) and CBF fluctuations within the arteriole and venule identified in **e**. Data presented is from a single animal and scale bar indicates 500 μm. Also see Supplementary Table 2

Most in vivo neuroimaging experiments in rodents are conducted under general anesthesia^39,40^. In many cases, a single injection (e.g., a ketamine/xylazine cocktail) is used to provide anesthesia for the entire duration of the experiment^41,42^. Once the depth of anesthesia is confirmed (e.g., by toe-pinching), it is usually assumed that baseline brain physiology remains stable and that observed variations are attributable to the experimental paradigm. We used the multi-contrast capability of our microscope to assess the validity of this assumption. Following anesthesia with ketamine/xylazine, we interrogated CBF changes (with LSC) over a 3 × 3 mm^2^ FoV in the right hemisphere of the mouse brain (Fig. 5a, see Methods). An EEG electrode implanted on the contralateral hemisphere, allowed concurrent global electrophysiological monitoring via an external EEG recording instrument (Fig. 5b, see Methods). Both CBF and EEG power continually decreased during deep anesthesia (Fig. 5b, ~20%). At the end of an hour, anesthesia was re-confirmed by the lack of response to a hind-paw pinch. Signs of wakefulness (e.g., first twitching of whiskers, then body movement etc.) emerged at around two hours. Both CBF and EEG power reached their minimum by this time, and increased with improving signs of wakefulness (Fig. 5b). Changes in total EEG power correlated with changes in CBF (*R*^2^ = 0.70). The power in standard EEG sub-bands showed fluctuations typical of the transition from the sleep to wakefulness states^43^, and did not exhibit any correlations with CBF (Fig. 5c).

During the same experiment, we also used the fluorescence channel to differentiate the microvascular bed into its arterial and venous components by exploiting the arrival times of an intravenously injected fluorescent tracer (dextran-FITC, 70 kDa). (Fig. 5d, see Methods and Supplementary Movie 6). We then identified an arteriole and a venule (Fig. 5e), and tracked changes in their blood flow (with LSC) and blood volume (with 570 nm IOS). The arteriole exhibited tight coupling between blood volume and blood flow (Fig. 5f); namely initial vasoconstriction and decreased blood flow followed by eventual vasodilation and recovery in blood flow, suggesting vessel diameter-based flow modulation. However, this relationship was different for the venule that exhibited blood flow alterations uncorrelated with changes in blood volume (Fig. 5g). While additional experiments are necessary for exploring the relationship between CBF regulation and electrophysiology under the influence of anesthetics, these preliminary observations suggest that global and local variations in brain physiology at the microvascular scale may occur during the anesthetized state, as well as the transition to the awake state.

### Life-cycle brain tumor imaging reveals daily angiogenesis-induced changes

**Fig. 6:**
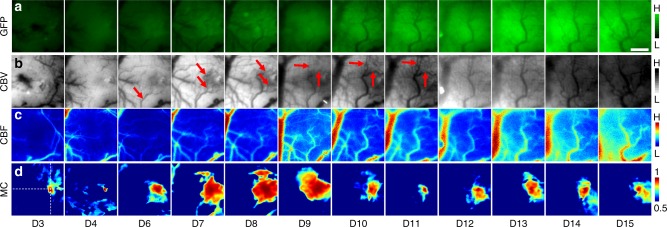
Life-cycle brain tumor imaging reveals angiogenesis-induced changes. Panels illustrating (**a**) brain tumor progression (GFP), and concomitant changes in (**b**) cerebral blood volume (CBV) and (**c**) cerebral blood flow (CBF) due to tumor angiogenesis. **d** Microvascular connectivity (MC) changes were also mapped in the freely behaving animal over the entire life-cycle (i.e., 16 day period after brain tumor inoculation on D0) of the brain tumor. MC was calculated by correlating CBV fluctuations from the entire FoV over 5 min with those from the seed pixel indicated by crosshairs. Data presented is from a single animal and scale bar indicates 500 μm

To demonstrate its utility in probing preclinical disease models over their entire life-cycle, we used our microscope to characterize structural and functional cerebrovascular changes during brain tumor progression (Fig. 6). In a freely behaving animal, we successfully mapped tumor extent via fluorescence imaging of GFP-expressing brain tumor cells (Fig. 6a), and observed an increase in GFP fluorescence, as well as expansion of the overall fluorescing area with brain tumor progression. This illustrates the capability of the microscope to longitudinally track increases in cancer cell density and tumor burden. Additionally, we continuously imaged the dynamic cerebrovascular landscape over 16 days (Fig. 6b) in terms of its structural and functional alterations. We initially observed the formation of a new mother vessel (Fig. 6b D6, red arrow) migrating toward the tumor inoculation site. Subsequently, we observed angiogenic sprouting from this parental vessel to support tumor growth (Fig. 6b, D6-D11, red arrows). To determine whether newly formed microvessels were functional, i.e., whether they were patent and carried flowing blood, we imaged the same ROI with LSC. The corresponding CBF maps (Fig. 6c) demonstrate that perfusion followed the vessel sprouting stage, i.e., some microvessels that were visible in the CBV images at D6 were not visible in the corresponding CBF images until D7 or D8. Finally, we assessed correlations between hemodynamic fluctuations^44^, which we dubbed microvascular connectivity (MC) in the tumor affected ROI (Fig. 6d) using IOS images continuously acquired while the animal was awake and freely moving. We observed an expanding region of microvascular connectivity from D6 to D9 that corresponded to the establishment of a de novo microvascular territory via tumor angiogenesis (Fig. 6b) followed by eventual perfusion stabilization (Fig. 6c). These observations collectively demonstrate the utility of a miniature multi-contrast microscope such as ours for interrogating preclinical disease models such as brain tumors, over their entire life-cycle.

### Rapid prototyped high-magnification adapter reveals erythrocyte flux

**Fig. 7:**
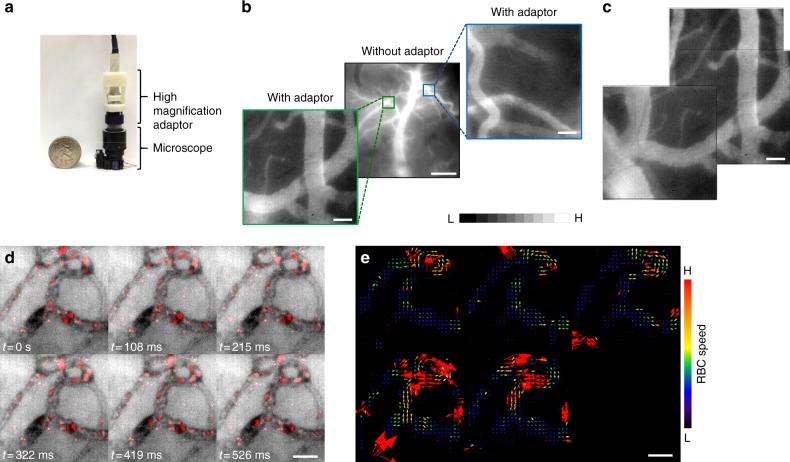
3D printed high-magnification adapter reveals microvascular hemodynamics. **a** The microscope is shown with the 3D printed high-magnification adaptor alongside a U.S. quarter coin for scale. **b** Image of fluorescent dye enhanced cortical vasculature ROI. Insets are magnified fields of view from this ROI. **c** High-magnification mosaic stitched together from three different fields of view. Images in **b**, **c** are normalized to 1% of their intensity ranges. **d** Video frames from a fluorescent-dye enhanced vessel plexus from the mouse ear in which the RBCs have been highlighted in red. One can clearly visualize the motion of individual RBCs over time in each vessel segment, from which one can conduct particle velocimetry and compute the direction and magnitude of RBC flux as shown in **e**. Cortical and ear microvascular data presented are from two different animals, respectively. Scale bar indicates 50 μm, except in **b**: where it is 500 μm. Arrows indicate vessel sprouting during D6-D11. Also see Supplementary Movie 7

To demonstrate the versatility of the miniaturized microscope in imaging microvascular hemodynamics, we 3D printed a single lens magnification adapter (Fig. 7a) that increased the spatial resolution from 5 to 0.5 µm (Fig. 7b). This modification permitted the generation of high-resolution image mosaics of the microvascular bed (Fig. 7c). Finally, in the mouse ear we demonstrated the microscope’s ability to acquire video rate movies of red blood cell (RBC) flux within individual vessels (Fig. 7d and Supplementary Movie 7) and successfully conducted particle velocimetry on the blood flow within these microvessels by tracking the trajectories of individual RBCs (Fig. 7e, see Methods).

## Discussion

The emerging role of the neurovascular unit, cerebrovascular remodeling and hemodynamic changes in neuropathologies ranging from Alzheimer’s disease to brain cancer necessitates the development of new tools for elucidating their role in preclinical models of these diseases^45,46^. Moreover, there is an exigent need for imaging tools capable of simultaneously interrogating multiple physiological variables ranging from neural activity to hemodynamics, while being synchronized with external electrophysiological monitoring equipment, and without the confounding effect of anesthetics. Therefore, in this work we developed a miniaturized microscope capable of in vivo neurovascular imaging in awake freely moving animals and demonstrated its utility by: (i) using multi-contrast imaging to characterize neurovascular changes that accompany auditory stimulation; (ii) performing wide-area functional brain mapping analogous to functional MRI; (iii) correlating hemodynamic and electrophysiological changes using EEG synchronized multi-contrast imaging during recovery from anesthesia, and (iv) characterizing changes in microvascular connectivity over the entire life-cycle of a preclinical brain tumor model.

Miniaturization has enabled scientists to head-mount tiny microscopes on awake and freely moving rodents for in vivo neuroimaging without the confounding effects of anesthetics^5^. However, most miniature microscopes are designed for conducting either neural or hemodynamic imaging, but not both (Please see Supplementary Table 1 for a comparison of current miniaturized microscopes). To overcome this limitation, we used 3D printing technologies and state-of-the-art miniature opto-electronic components to shrink the dimensions of a conventional multi-contrast neuroimaging system^13,20^ from several feet (e.g., 5 × 5 × 5 feet^3^) down to the size of an adult human fingertip (5 cm^3^) (Figs. 1–2). In addition, only a laptop was required to power and control this microscope, ensuring complete portability of the imaging system. Finally, our miniaturized microscope offers plug-and-play functionality, permits real-time imaging and control of all the imaging parameters via a user friendly GUI.

We exploited the microscope’s high spatial (5 μm) and temporal (15 fps) resolution, and its 3 × 3 mm^2^ FoV to image the entire auditory cortex at microvascular spatial resolution in an awake mouse. We observed how neural calcium dynamics, vasodilation of individual microvessels and local CBF levels were modulated by an auditory stimulus. We employed a specialized GLM approach that exploits the microscope’s multi-contrast capabilities to remove background fluctuations from each hemodynamic response variable. This permitted the identification of more localized hemodynamic responses in contrast to conventional global hemodynamic mapping approaches^18^. We were able to successfully demonstrate the well-known tonotopy of the murine auditory cortex using the hemodynamic responses to 4 and 24 kHz sound stimuli^47,48^ and the spatially localized GCaMP signal as the gold standard for tonotopic neural activity.

Next, we used the microscope’s sync channel to time-lock image acquisition with EEG recording. This feature is unavailable in the current crop of miniaturized microscopes, and permitted us to correlate global CBF changes with synchronized EEG recordings during the animal’s recovery from the anesthetized state. In addition to demonstrating the recovery of CBF from ketamine-xylazine observed by others^49^, we also found a strong correlation between global CBF and EEG power. This observation is consistent with other reports characterizing the relationship between CBF and EEG^50,51^. In contrast, the power changes within EEG sub-bands did not correlate with global CBF changes, but showed the classic transition into the awake state (i.e., reduction in low frequency power, and increase in the power at higher frequencies^43,52^). We also used the microscope’s fluorescence channel to discriminate arteries from veins by measuring arrival times of an intravenously injected fluorescent dye. We then used this information to demonstrate that arterioles and venules can exhibit unique flow-volume relationships that are modulated during anesthesia, as well as during the transition to the awake state. Collectively, these proof-of-principle experiments showcase the microscope’s ability to map multiple global and local signal changes in anesthetized and awake animals.

Finally, we used our miniature microscope to assess angiogenesis-induced cerebrovascular remodeling over the entire life-cycle of a brain tumor. The multi-contrast capability of our microscope enabled us to track tumor growth (via FL), the formation of angiogenic microvessels to support such growth (via IOS), and the establishment of perfusion in those vessels two days later (via LSC). Next, we used fluctuations in the IOS signal acquired from the freely moving animal to map alterations in microvascular connectivity due to tumor progression. Unlike conventional functional connectivity measures used in MRI, microvascular connectivity is the direct mapping of correlations between CBV fluctuations from individual blood vessels made possible by the high spatio-temporal resolution of the miniaturized microscope. Here we used it as an index of cerebrovascular functionality rather than a surrogate of underlying neural activity. We found that in the absence of well-established blood vessels (D3-D4) there was minimal microvascular connectivity. Next, we observed increased microvascular connectivity (D6-D9), consistent with brain tumor-induced angiogenesis and the establishment of anastomoses within the angiogenic vessel bed^46^. Finally, we observed the stabilization of microvascular connectivity during the final week (D10-D15) of tumor progression before the animal succumbed to its tumor burden. To the best of our knowledge, this is the first time a miniaturized multi-contrast microscope has been used to longitudinally track disease burden and disease-induced changes of multiple physiological variables in an unanesthetized animal, over the entire life-cycle of the disease model. This approach could easily be adapted for functional imaging in preclinical models of neurodegenerative diseases (e.g., Alzheimer’s disease), as well as conditions such as stroke.

This microscope represents a significant advance in design and miniaturized fabrication. In contrast to other miniaturized microscope designs^7,22^, we were able to achieve wider cortical coverage by relaxing the magnification requirement. For example, the miniature microscope described in Ghosh et al^7^ achieved a magnification of ×5, but its FoV was limited to ~11% of that achievable with the current microscope. This wide-field capability is essential when interrogating complete cortical regions (e.g., auditory cortex) or analyzing the functional overlap between adjacent cortical regions (e.g., auditory-somatosensory cortices). Finally, this design modification made it possible to accommodate three optical contrast mechanisms for characterizing multiple neurophysiological variables with the same microscope (please see Supplementary Table 1 for a comparison of current miniaturized microscopes).

The multi-contrast capability of our microscope enabled us to interrogate neurovascular variables critical to neuroscientific and neuropathological investigations. These capabilities included FL, IOS, and LSC imaging. As shown by other studies, the inclusion of a fluorescence channel facilitates imaging of neural activity^53^, astrocytes^54^, fluorescently tagged cells^55^, as well as potentially the uptake of fluorescently labeled pharmacological agents. The availability of separate IOS and LSC imaging channels enables the independent characterization of microvascular morphology and blood flow. This is especially useful in applications in which the regulation of blood flow and vessel architecture is dysregulated (e.g., during tumor angiogenesis^56^), or when the microvascular network is poorly perfused (e.g., during stroke^57^). Concurrent assessment of dHb absorption under red light illumination can supplement measurements from the other contrast mechanisms yielding vital information on oxygenation status and could help identify hypoxic regions (e.g., ischemia^58^, tumor necrosis^59^) and hyperoxic regions (e.g., functional activation^14^). The ability to map dHb could also be exploited to directly validate BOLD fMRI data with optical imaging data^15^. Correlating multi-contrast data from our microscope with behavioral observations in freely moving animals would add another dimension to current neurophysiological studies. For example, with our head-mounted microscope one could conduct in vivo imaging of cognitive activity while the mouse is exploring a maze. Finally, rapid prototyping a magnification adapter permitted this microscope to acquire images over spatial resolutions ranging from the entire auditory cortex (5 µm) down to individual cortical microvessels (~0.5 µm).

We also showcased the use of a non-standard image-processing pipeline (i.e., gold-standard based GLM analysis) on high-resolution multi-contrast optical imaging data. In contrast to the convention of showing a single optical time-course from an activated region or highlighting activations based on a single contrast mechanism^36,37^, we demonstrated the ability to conduct wide-area mapping of neural activity and the accompanying hemodynamic response. Moreover, this multi-contrast GLM-based approach wherein neural and hemodynamic responses could be characterized simultaneously and independently, enhanced the spatial localization of the hemodynamic response compared to conventional methods. Such multi-contrast GLM-based processing schemes have the potential to improve and inform image-processing strategies for other optical imaging or fMRI studies. Our demonstration serves as a model for microscopy image processing, and provides a data analysis framework for other researchers using such approaches.

Our microscope offers all these benefits at a fraction of the cost of a multi-contrast benchtop imaging system. This is due to our extensive use of 3D printing technologies and materials that are more economical than conventional machining/fabrication, as well as our use of commercially available miniature opto-electronic components. The disposable head mount and flexible microscope attachments allowed the microscope to be removed from the animal when imaging was not being conducted. This plug-and-play feature enables our microscope to be used concomitantly on multi-animal cohorts wherein longitudinal (e.g., daily) in vivo imaging is necessary. We believe in the near future this microscope’s affordability and flexibility will play a major role in making it a ubiquitous laboratory tool.

We envision several exciting additions to the microscope that could enhance its extant capabilities. For example, the use of a multi-wavelength laser diode^60^ instead of a single wavelength laser would enhance the estimation of oxygen saturation. The addition of an LED for optogenetic stimulation^61^ could equip our microscope with the capacity to excite or inhibit neural activity while performing concurrent multi-contrast imaging. In addition, the cranial window preparation through which imaging is carried out could be modified to incorporate a transparent electrocorticographic (ECoG) grid^62^ to allow direct recording of neural activity. Simultaneously recording electrophysiology along with blood flow^51^ or oxygen saturation estimation would be very useful in imaging stroke and its penumbra region^63^, or imaging areas of spreading depression^63^, or areas spawning epileptogenic response to focal drug injection^20^. With its current capabilities and potential for expandability, we believe our microscope will herald an exciting new era in neuroscience research.

## Methods

### Head mount design

Figure 1d–e shows bottom and side views of the head mount. The head mount is implanted on a surgically exposed rodent skull, and enables our microscope to firmly attach via a pair of locking screws. The prong structure on the bottom of the head mount allows it to be centered on the FoV at the time of implantation. A different head mount design was used for the auditory stimulus experiment because it required attachment of the microscope to a head-immobilized mouse (Supplementary Fig. 5).

### Illumination control module

We designed a compact illumination control module to deliver user-programmable levels of illumination (Supplementary Fig. 2). A microcontroller (Uno, Arduino.cc, Turin, Italy) receives commands from the master program residing on the PC regarding the ON/OFF status of each illumination source, as well as the current to be delivered to each. If LED illumination is desired, the microcontroller activates one of its pulse width modulation (PWM) modules. The duty cycle to be used was calculated by the program running on the microcontroller. Each of the blue, left and right green LEDs have a dedicated PWM module allowing flexible illumination control. Since the laser diode (Vixar Inc., MN) required a continuous current, a digital-to-analog (D/A) module was used to create an analog voltage level that was converted into a corresponding current level by a voltage-to-current converter circuit. Current levels of up to 5 mA were used to drive the blue LED, while the green LEDs each required ~20 mA. The laser diode was driven at 2–2.5 mA.

### System architecture

The overall system architecture is shown in Supplementary Fig. 3a. A customized master program residing on a laptop PC controlled all data and command flow. The commands received by the illumination controller result in ON/OFF switching of different illumination sources. Additionally, the master program communicated with the image acquisition module (Awaiba, Idule module) to set the exposure time of the image sensor, as well as initiate image acquisition. Images were then sent via the image acquisition module to the master program that stored the data on external memory storage. Image processing was conducted off-line.

### Strain relief mechanism

A suspension mechanism comprising elastic bands was used to reduce the head-borne weight of the microscope (Supplementary Fig. 1a). This suspension mechanism had a spring constant of 1 g/4 mm. Therefore, for a maximum vertical head displacement of 1 cm, the resulting weight on the animal’s head was 3 g. Via behavioral videos we also verified that the head-mounted microscope did not impede the animal’s natural behaviors (Supplementary Movie 1). Finally, we assayed blood corticosterone levels in animals over two weeks to demonstrate that the head-mounted microscope did not induce additional stress during imaging (Supplementary Fig. 1b).

### Cranial window preparations

All animal procedures were approved by the Johns Hopkins Animal Care and Use Committee. For microscope validation, anesthesia recovery and high-magnification adaptor experiments, anesthesia was induced via an intraperitoneal injection of ketamine (90 mg/kg) and xylazine (5 mg/kg). After checking the depth of anesthesia with manual forepaw or tail stimulation (pinching), eye ointment (Puralube, Dechra Veterinary Products, KS) was spread over the mouse’s eyes to prevent dehydration. The fur over the scalp was trimmed and the exposed area sterilized with 70% ethanol. An incision was made over the right parietal bone and the overlying skin and fascia were removed. After thoroughly drying the exposed area, the boundary of the cranial window to be drilled was marked with a pen. Next, the skull was thinned with a micro-drill (Aseptico Inc., WA) until the underlying vasculature became visible. The entire area was flushed with saline and thoroughly dried. The head mount was centered on the cranial window via the prongs and attached using a thin layer of cyanoacrylate glue (Loctite, Henkel AG & Company KGaA, Düsseldorf, Germany). A self-curing dental cement (Parkell Inc., NY) was used to secure the head mount to the skull and cover any exposed areas (Fig. 1f).

For the auditory stimulus experiment, anesthesia was induced with 4% isoflurane at an O_2_ flow rate of 0.5 L/min. The mouse head was secured to a stereotaxic frame with a bite bar while anesthesia was maintained at 1–2% isoflurane with 0.5 L/min O_2_ flow rate through a tube attached to the bite bar. Petroleum jelly (Vaseline, Unilever, NJ) was spread over the mouse’s eyes to prevent dehydration. The hair over the scalp was trimmed and the exposed area sterilized with 70% ethanol. Injections of dexamethasone (2 mg/kg) and carpofren (5 mg/kg) were administered. After lidocaine (2% with 1:100,000 epinephrine) was injected locally, an incision was made on the midline of the head. The skin and fascia were removed over the area of the cranial window. The area was thoroughly dried and a dental bonding agent (Optibond, Kerr Corp., CA) applied to the surface. A custom-built steel head post was secured with a UV cured dental cement (Heraeus Kulzer GmbH, Hanau, Germany) to the exposed area of the skull. Using the underlying vasculature and local landmarks, the area of the cranial window (above the left auditory cortex) was identified with a pen. A craniotomy was performed with a micro-drill (Foredom Electronics Co., CT) and the bone detached from the skull while leaving the dura intact. After clearing the area of debris, a cover-slip was placed on top of the craniotomy and secured with self-curing dental cement (Parkell Inc., NY). During the procedure, 0.9% saline was injected to prevent dehydration. After the procedure was completed, buprenorphine (0.5 mg/kg) was injected and bacitracin was administered topically (Supplementary Fig. 5d).

For the brain tumor life-cycle imaging experiment, a similar procedure as described above was performed, except for the attachment of the steel head post. Before placement of the glass coverslip, 100,000 9L-GFP brain tumor cells were inoculated at a depth of 2 mm near the center of the cranial window (i.e., 2 mm anterior to bregma, 2 mm right of the mid-line) followed by attachment of the microscope head-mount. Parental 9 L brain tumor cells were obtained from the Hunterian Neurosurgical Laboratory (B.M.T.) at the Johns Hopkins University School of Medicine and transfected with an EGFP-expressing plasmid to obtain stably expressing 9L-GFP cells. All cell lines tested negative for mycoplasma contamination.

### Brain imaging procedures

For microscope validation, a thinned-skull cranial window was prepared on a female C57BL/6 mouse as described above. After recovery, the mouse was anesthetized briefly with isoflurane in a chamber. Subsequently, anesthesia was maintained through a nose cone by providing 2% isoflurane mixed with room air at 1 L/min flow rate. An oxygen inhalation challenge was performed under red laser illumination: 30 s of oxygen was provided after a 2 min baseline of room air. Imaging was continued for a total of 4 mins. Next, a stack of images was acquired under green light illumination. For fluorescence imaging, 0.2 mL of 10 mg/mL dextran-FTIC (70 kDa) was injected via the tail vein. Images were acquired under blue (i.e., 452 nm) light illumination before and after the injection. The mouse was then moved to the benchtop imaging system, and the same imaging protocols performed. The benchtop imaging system used a 632 nm laser for laser speckle illumination, a white light source with a 470 ± 5 nm filter for green light illumination, as well as a 610 ± 5 nm filter for red light illumination. A 473 nm laser was used for blue light illumination/FITC excitation, and a 496 nm long-pass filter was used for filtering the FITC emission.

For the auditory stimulus experiment, a craniotomy-based optical window was prepared over the left auditory cortex of a female tetO-GCaMPs×CaMKII-tTA mouse as described above^32^. After complete recovery from surgery and habituation to the imaging setup, the mouse was briefly anesthetized by isoflurane inhalation in a chamber, and subsequently its head immobilized in the imaging apparatus. The mouse remained awake during the entire experiment. Imaging runs were split into sixty epochs of 10 s each (i.e., total imaging duration per run = 10 min) and images acquired under blue light illumination with an exposure time of 50 ms at ~15 fps. Within each epoch, a 300 ms auditory stimulus was presented at the 3 s time point. The auditory stimulus was randomized to be either 4 kHz or 24 kHz. A total of 30 stimulus presentations for each 4 kHz and 24 kHz were conducted. This procedure was then repeated under green light and red laser diode illumination. Imaging under green light and red laser diode illumination was conducted at 50 ms and 10 ms exposure times, respectively. The frame rate remained ~15 fps. The aural stimulus generator output an electrical pulse that activated the sync channel LED at the beginning of each 10 min trial. This enabled the microscope data and the stimuli to be synchronized. Each image acquired by the microscope was time-stamped at ms temporal resolution.

For the recovery from anesthesia experiment, a thinned-skull cranial window was prepared on a female SCID mouse as described above. The animal was anesthetized with 0.45 mL of ketamine/xylazine within the scope of 2 h needed for the surgery, microscope fixation and the administration of dextran-FITC (0.2 mL, 10 mg/mL, 70 kDa). Images were continuously acquired under blue light illumination (50 ms, ~15 fps) during dye injection. Then, for the next 200 min until the mouse showed signs of being fully awake, a stack of 100 images under red laser diode illumination (5 ms exposure time) and 10 images under green light illumination (100 ms exposure time) were acquired every minute.

For the brain tumor life-cycle imaging experiment, a craniotomy-based optical window was prepared at the time of tumor inoculation on a female athymic nude mouse. Imaging was performed each day over a 16 day period before euthanasia. After briefly anesthetizing the mouse with isoflurane to mount the microscope, the animal was permitted to awaken and 5 min of resting-state data were acquired at ~15 fps under green light and red laser diode illumination. The microscope was then unplugged after another brief period of isoflurane induced anesthesia. In addition to resting state imaging data, a 1 s burst of fluorescence images was acquired under blue light illumination to map tumor extent via GFP expression of tumor cells.

For the high-magnification adaptor experiment, a thinned-skull cranial window was created on a female SCID mouse as described above. After recovery, the mouse was anesthetized briefly with isoflurane. Subsequently, anesthesia was maintained through a nose cone by providing 2% isoflurane mixed with room air at 1 L/min flow rate. 0.2 mL of 75 mg/mL dextran-FITC (70 kDa) was injected through the tail vein. Images were captured under blue light illumination after the injection, with and without the high-magnification adaptor, to enable comparison of the same FoV at multiple spatial resolutions.

### Ear imaging procedures

A female athymic nude mouse was anesthetized briefly with isoflurane in a chamber and its ear placed in a custom-built optically transparent holder. Subsequently, anesthesia was maintained through a nose cone by providing 2% isoflurane mixed with room air at 1 L/min flow rate. Dextran-FITC (0.2 mL, 75 mg/mL, 70 kDa) was administered via the tail vein and images continuously acquired under blue light illumination (100 ms, ~8 fps) after dye injection.

### Image processing

For all experiments, the HbT and dHb levels were calculated by inverting IOS images acquired under green light and red laser diode illumination, respectively. Next, LSC was calculated by processing a stack of red laser images. The speckle contrast (*k*) at each pixel (*x*, *y*) is calculated as:^29^

1 $$k\left( {x,y} \right) = \frac{{\sigma (x,y)}}{{{\mathrm{\mu }}(x,y)}}$$

Here, *µ* and *σ* are the mean and standard deviation of light intensity at each pixel in the chosen image stack.

The speckle contrast (*k*) is related to blood flow by:^29^

2 $$k^2 = \frac{\tau }{{2T}}\left\{ {2 - \frac{\tau }{T}\left[ {1 - \exp \left( { - \frac{{2T}}{\tau }} \right)} \right]} \right\}$$

Here exposure time is denoted by *T* and *τ* denotes the decorrelation coefficient, a quantity inversely proportional to blood flow. For small *τ* values (associated with typical microcirculation imaged by our microscope), Eq. 2 can be simplified to:

3 $$\frac{1}{\tau } \propto \frac{1}{{k^2}}$$

Equation 3 was used to compute CBF.

For microscope validation, benchtop images were co-registered to microscope images by performing a landmark-based image registration (ImageJ^64^, TurboReg) followed by 5 × 5 pixel median filtering and background signal removal by applying a background subtraction filter in ImageJ that was 50 pixels in radius. The pre-injection fluorescence image acquired by the microscope was subtracted from the post-injection fluorescence image to enhance the contrast-to-noise ratio. Next, a 20 × 20 pixel grid superimposed over a central 400 × 400 pixel FoV was used to calculate the correlation coefficients between the microscope and benchtop acquired FL, IOS, and LSC images. Additionally, the correlation between the 680 nm IOS image time-course acquired with the microscope and the 610 nm IOS image time-course acquired with the benchtop imaging system was calculated during the oxygen inhalation challenge, over a central 20 × 20 pixel sub-region of the FoV.

For the auditory stimulus experiment, images from 30 trials for each stimulus presentation were averaged to calculate activation maps. One trial each under green light illumination and red laser diode illumination was excluded due to noise. Since the image sensor frame rate can vary, each image was time-stamped at ms temporal resolution. This permitted the acquired images to be resampled with a temporal resolution of 10 ms (Matlab) using linear interpolation. Next, a 10 s long average response image stack was calculated for each contrast mechanism. A 1 s moving average temporal filter was applied to reduce noise limiting the useful range from 1–10 s. Calcium and hemodynamic responses were calculated using ImageJ according to:

4 $$r\left( t \right) = \frac{{x\left( t \right) - \bar x_B}}{{\bar x_B}}$$

Here, *r*(*t*) is the calculated response, *x*(*t*) the measured signal corrected for illumination variations (i.e., GCaMP bound calcium fluorescence, HbT, CBF or dHb), and $\bar x_B$ the mean pre-stimulus signal (i.e., 1–3 s). Illumination variations were estimated by applying a 100 × 100 pixel mean filter to the IOS image acquired during the pre-stimulus period for each IOS modality. After computing *r*(*t*), a 3 × 3 median filter was applied to reduce noise. This translated into an effective spatial resolution of 15 µm. The laser illumination based data (dHb and CBF) underwent additional filtering with a 20 × 20 pixel mean filter.

For wide area functional mapping analogous to classic task-based fMRI processing, the overall signal processing model is schematically illustrated in Fig. 4a. Here, the stimulus-specific hemodynamic response function (i.e., the idealized response), and the cortex wide hemodynamic fluctuation (i.e., the background signal) were used as factors in a general linear model^65^ (GLM) to fit the observed signal and compute activation and background fit coefficients on a pixel-wise basis. An error term was also included in the GLM.

The hemodynamic data was filtered in the temporal domain using a low-pass filter with a 2 Hz cut-off for noise removal. Next, as shown in Supplementary Fig. 4, a hot-spot time course was created by taking the average hemodynamic time-course for a region exhibiting peak neural activity (identified as a hot-spot in the maximum intensity projection image of the calcium response). The background signal was computed by taking the cortex-wide average. This background signal was then subtracted from the hot-spot time-course to produce the idealized response. The filtered hemodynamic data, together with the idealized response and the background signal was input to a GLM as shown below (Fig. 4a):

5 $$r\left( t \right) = c_0 + c_1a\left( t \right) + c_2g\left( t \right) + e(t)$$

Here, *a*(*t*) and *g*(*t*) are the idealized response and the background signal, respectively. *c*_1_ and *c*_2_ are their corresponding fit coefficients computed by the model. *r*(*t*) is the observed hemodynamic time course, *e*(*t*) is the error term and *c*_0_ is an offset adjusting constant. This pipeline was performed separately for CBF and HbT hemodynamics. The entire procedure was first performed for 4 kHz stimulus data and then repeated for the 24 kHz stimulus data.

For the anesthesia recovery experiment, HbT and CBF levels of individual vessel segments were analyzed after removing the effect of the background signals by applying a background subtraction filter in ImageJ that was 50 pixels in radius. Additionally, each HbT image was corrected for illumination intensity fluctuations that were estimated by applying a 200 × 200 pixel mean filter to the corresponding IOS image. An artery and a vein ROI (30 × 30 pixels each) were then selected for hemodynamic analyses.

To calculate arrival time of the injected fluorescent tracer in each pixel, the acquired fluorescence image stack was first resampled to 50 ms temporal resolution. Next, a background subtraction filter (ImageJ, 50 pixel radius) and a low pass filter (2 Hz cutoff) were applied to remove background fluctuations and high frequency noise, respectively. Then, a single intensity threshold was applied for detecting the fluorescent tracer within the FoV. Arrival times were calculated as the time taken for the fluorescent intensity in each pixel to reach its threshold value and a 5 × 5 pixel median filter was applied to reduce noise. Finally, a binary vessel mask was applied to segment the cerebral vessels and  a map of arrival times generated and scaled  for visualization purposes.

To calculate microvascular connectivity (MC) for the brain tumor life-cycle imaging experiment, the HbT image time-series was averaged into 1 s blocks and band limited to 0.01–0.1 Hz. Next, global fluctuations were estimated and eliminated using linear regression^66^. Finally, MC was calculated as the correlation coefficient between the time course of a seed pixel (as shown in Fig. 6) and that of each pixel within the FoV.

For the high-magnification adaptor experiment, to identify the RBC aggregates within each video frame we inverted each image, corrected the background for non-uniform illumination, followed by contrast enhancement. Next, we computed the largest and middle eigenvalue images of the Hessian tensor using the FeatureJ plugin in ImageJ, combined them with a max operator (i.e., img1 = max (img1, img2)), thresholded the result and overlaid the resulting image of RBCs (in the red channel) on the original grayscale video. This resulted in a movie in which the RBCs were highlighted in red. Quiver plots that show RBC flow speeds were calculated using the iterative PIV plugin for ImageJ. All images were processed using ImageJ.

### EEG signal processing

EEG was recorded at 12207 Hz (Tucker Davis Technologies, FL). We down-sampled the EEG data by a factor of 40 for subsequent processing (Matlab). Appropriate bandpass filters were implemented for the standard EEG frequency bands: delta, theta, alpha, beta and gamma^67^ (Supplementary Table 2). Additionally, to filter powerline noise, a 60 Hz notch-filter was implemented before passing the EEG data through the gamma filter. Next the power in each signal band was calculated by computing the filtered signal variance within 1 min epochs. A 3-sample median filter was used to reduce noise. Finally, total EEG power was computed as the sum of the power in each of the five frequency sub-bands.

### Code availability

Matlab code used in the manuscript will be made available to readers upon reasonable request from the corresponding author.

## Supplementary information

Supplementary Information

Description of Additional Supplementary Files

Supplementary Movie 1

Supplementary Movie 2

Supplementary Movie 3

Supplementary Movie 4

Supplementary Movie 5

Supplementary Movie 6

Supplementary Movie 7

Reporting Summary

## Data Availability

Study data will be made available to readers upon reasonable request from the corresponding author. A reporting summary for this article is also available as a Supplementary Information file.
