# Supplementary material for: A miniature multi-contrast microscope for functional imaging in freely behaving animals
Source: Nat Commun. 2019 Jan 9;10:99. doi: 10.1038/s41467-018-07926-z (PMC6327063; doi:10.1038/s41467-018-07926-z)
Supplement: Supplementary file 1 — Supplementary Information [file 41467_2018_7926_MOESM1_ESM.pdf]

A Miniature Multi-Contrast Microscope for Functional Imaging in Freely Behaving Animals

Senarathna et al.

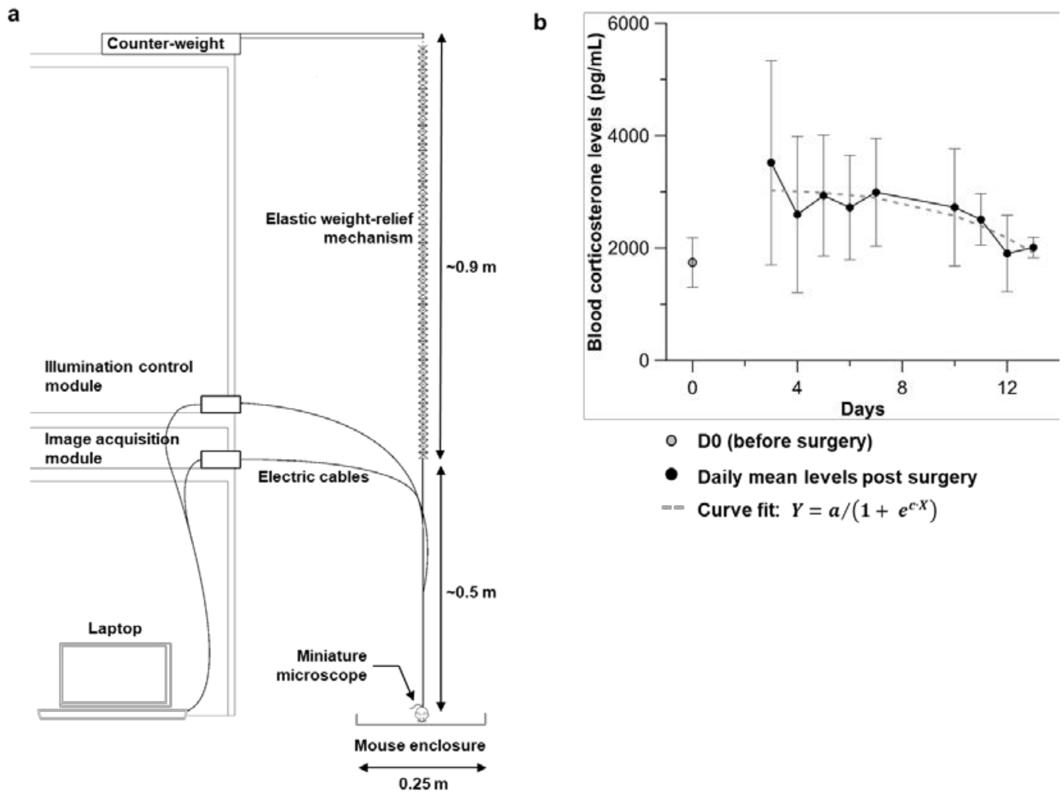

<u>Supplementary Figure 1: Weight-relief mechanism and mouse corticosterone levels</u>*.*

(a) Schematic of the weight-relief mechanism that comprised of interlocked elastic bands. At the equilibrium position, i.e. the average height at which we expect the mouse's head to be, the weight-relief mechanism bore the entire microscope weight (9g), enabling the mouse to behave naturally. We adjusted the spring constant of the mechanism so that only 1 g of weight was borne per 4 mm of head lift. Estimating head displacement to be within 10 mm of its natural head position, the mechanism reduced the microscope's head-borne weight to less than 3 g.

(b) To assess animal stress over 12 days of continuous microscope use we assayed blood corticosterone levels (mean ± standard deviation). Day zero (D0) was prior to microscope mounting. Cranial window surgery was performed on Day 3. Animals carried the microscope for 30-60 min/day, starting on Day 4. Blood samples were collected from Days 0-12. The average blood corticosterone level curve (n=3) and corresponding curve-fit (R^2=0.696) show the reduction in stress to the animal following habituation with the head mount and microscope.

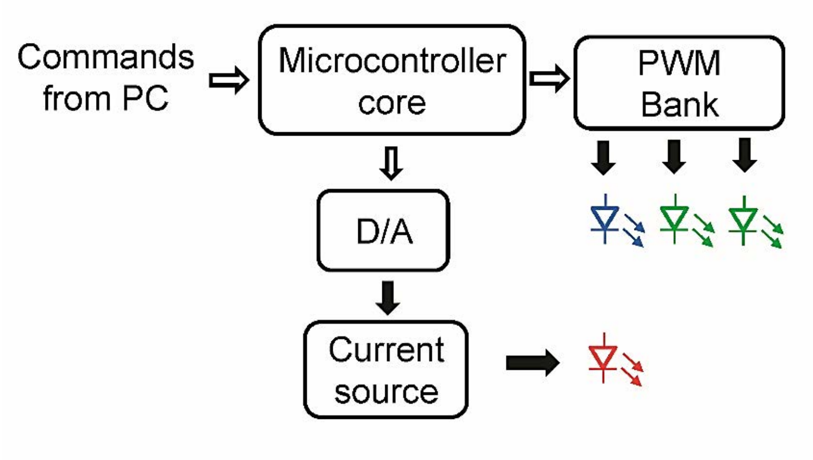

<u>Supplementary Figure 2: Schematic of the illumination control module</u>.

Commands from the master control program running on the PC were sent to the microcontroller core via serial communication, which then activated the PWM modules or the D/A converter. If LED illumination was desired, a combination of three separate PWM modules was activated: for the blue LED, and the left and right green LEDs. For laser illumination, the D/A converter created an analog voltage that was converted into a steady current level for the laser diode via the current source circuit. Digital transmission is indicated by hollow arrows and analog connections are indicated by solid arrows.

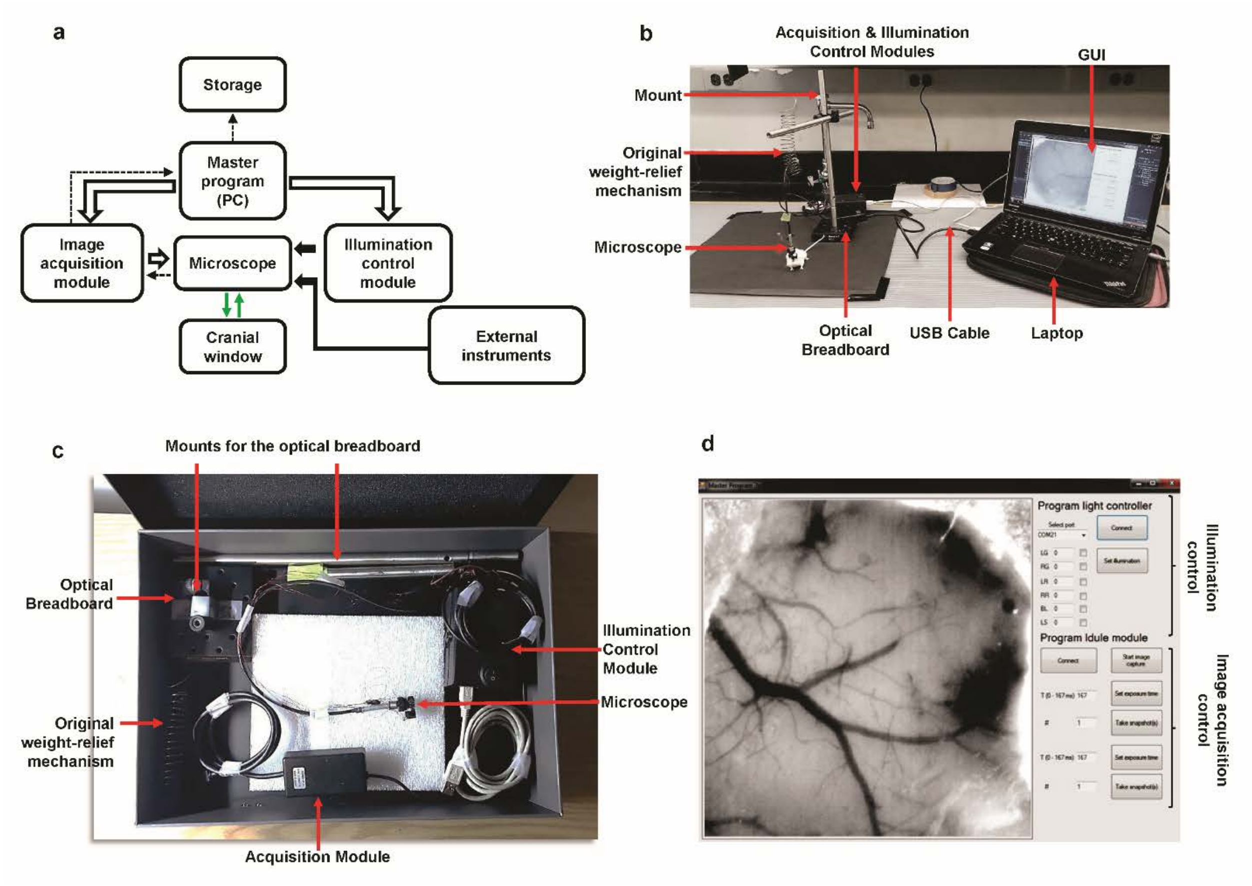

Supplementary Figure 3: Overview of the portable microscope system. (a) Schematic of the system architecture for controlling the microscope. A master control program running on a laptop PC acted as a hub for control and data flow. It sent digital commands via a USB interface to the illumination control module that switched the illumination sources. The master control program also sent commands to the image acquisition module that interacted with the image sensor. Acquired images were relayed via the image acquisition module to the master control program that then stored them. External instruments were synchronized with image acquisition via a synchronization (sync) channel. Digital communication is indicated by hollow arrows and analog currents by solid arrows. Illumination and light collection are represented by green arrows. Dashed arrows indicate the path of acquired images. (b) The system was controlled and powered by a laptop PC. (c) A 'briefcase' version of the microscope wherein all microscope parts could be stored for transport. (d) Screen shot of the GUI for controlling the microscope.

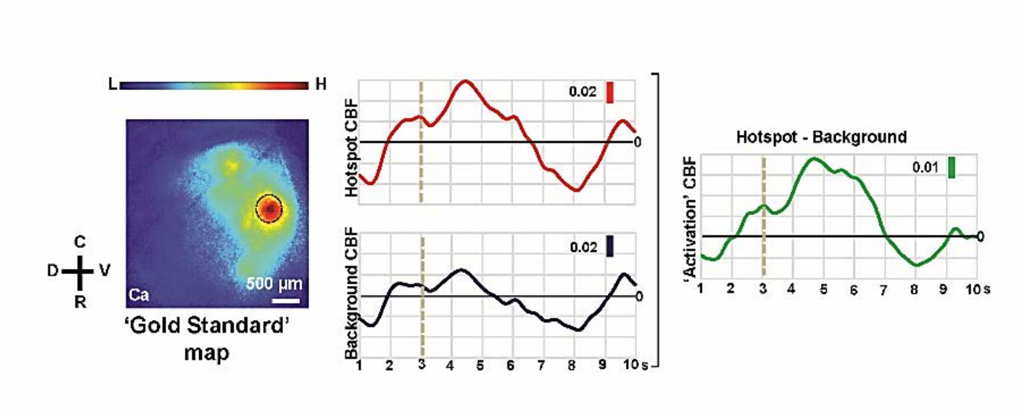

Supplementary Figure 4: Calculation of the activation hemodynamic response. The activation CBF response was computed by subtracting the average CBF time-series for the background from the average CBF time-series corresponding to the GCaMP hotspot (i.e. corresponding to the neural activation).

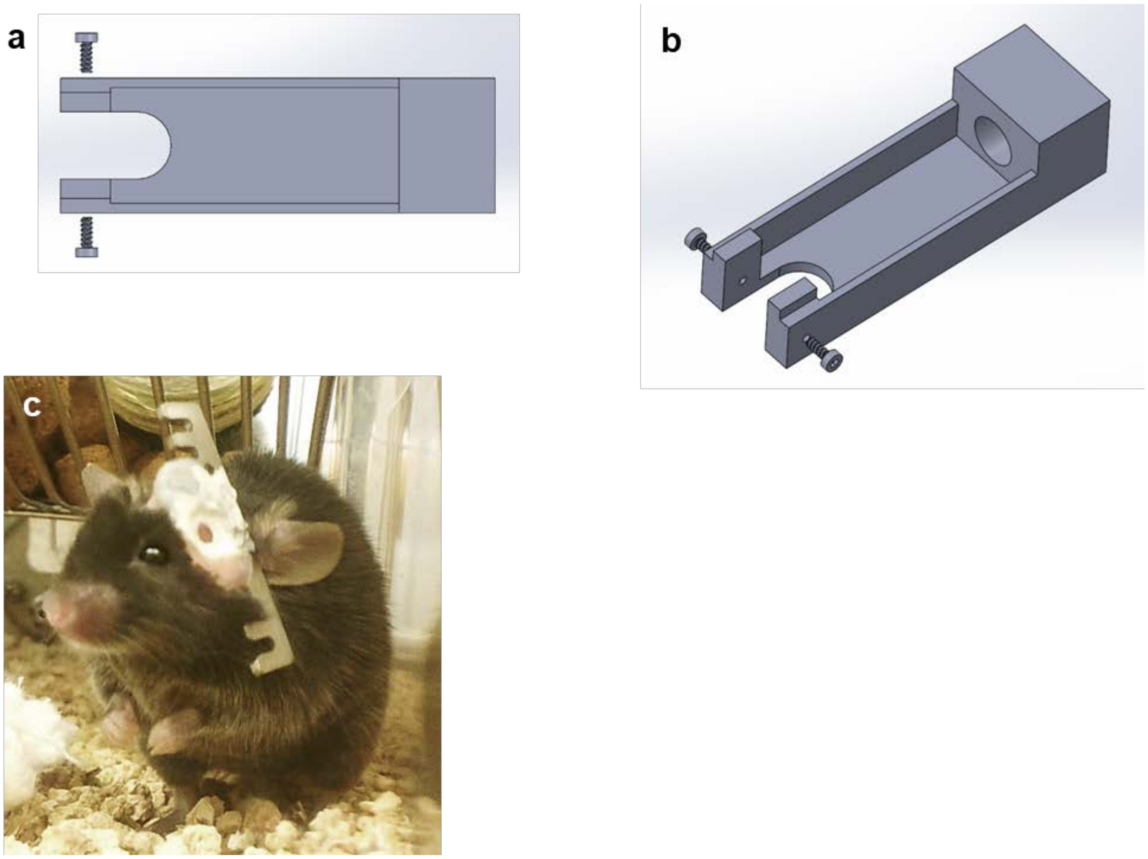

<u>Supplementary Figure 5: Experimental setup for the functional imaging experiment</u>.

(a, b) Top and side views of the head mount used in the auditory stimulus experiment. The head mount was attached firmly to an optical bread board via a bolt through a threaded hole. (c) A mouse bearing a steel head-post that mated with the head mount.

Supplementary Table 1: Miniature microscope comparison table.

Reference	Optical Technology	Physiologic Variable		
		Neural	HbT/Vessel Morphology/CBV	CBF
Engelbretch et al[1]	2P	✓		
Tang et al[2]	PAM		✓	✓
Xi et al[3]	OCT		✓	✓
Gosh et al[4]	FL	✓		
Park et al[5]	FL	✓		
Sigal et al[6]	IOS, LSC		✓	✓
Miao et al[7]	IOS, LSC		✓	✓
Current work	FL, IOS, LSC	✓	✓	✓

Scanning Technologies▶ 2P: Two-photon imaging; PAM: Photoacoustic Microscopy; OCT: Optical Coherence Tomography.

Wide-field Technologies▶FL: Fluorescence imaging; IOS: Intrinsic Optical Signal imaging; LSC: Laser Speckle Contrast imaging.

Supplementary Table 2: Filter Settings for Electrophysiology.

Band	Stop band 1 (Hz)	Pass band 1 (Hz)	Pass band 2 (Hz)	Stop band 2 (Hz)	Type	Order
Delta			4	5	Lowpass FIR	500
Theta	3	4	7	8	Bandpass FIR	500
Alpha	7	8	15	16	Bandpass FIR	500
Beta	15	16	31	32	Bandpass FIR	500
Notch	58	59	61	62	Bandstop FIR	500
Gamma	31	32	100	101	Bandpass FIR	500

Supplementary references

1. Engelbrecht, C. J., Johnston, R. S., Seibel, E. J. & Helmchen, F. Ultra-compact fiber-optic two-photon microscope for functional fluorescence imaging in vivo. *Opt. Express* 16, 5556-5564 (2008).

2. Tang, J., Dai, X. & Jiang, H. Wearable scanning photoacoustic brain imaging in behaving rats. *J. Biophotonics* **9**, 570-575 (2016)

3. Xi, J. *et al*. Diffractive catheter for ultrahigh-resolution spectral-domain volumetric OCT imaging. *Opt. Lett.* **39**, 2016-2019 (2014).

4. Ghosh, K. K. *et al*. Miniaturized integration of a fluorescence microscope. *Nat. Methods* **8**, 871-878 (2011).

5. Park, J. H. *et al*. Head-mountable high speed camera for optical neural recording. *J. Neurosci. Methods* **201**, 290-295 (2011).

6. Sigal, I. *et al*. Imaging brain activity during seizures in freely behaving rats using a miniature multi-modal imaging system. *Biomed. Opt. Express* **7**, 3596-3609 (2016).

7. Miao, P. *et al*. Chronic wide-field imaging of brain hemodynamics in behaving animals. *Biomed. Opt. Express* **8**, 436-445 (2017).
