# Supplementary material for: A miniature multi-contrast microscope for functional imaging in freely behaving animals
Source: Nat Commun. 2019 Jan 9;10:99. doi: 10.1038/s41467-018-07926-z (PMC6327063; doi:10.1038/s41467-018-07926-z)
Supplement: Supplementary file 2 — Description of Additional Supplementary Files [file 41467_2018_7926_MOESM2_ESM.pdf]

Description of Additional Supplementary Files

File Name: Supplementary Movie 1
Description: Freely behaving, unrestrained mouse with the headmounted miniature microscope (Related to Figure 1).

File Name: Supplementary Movie 2
Description: Wide-area Ca2+ response (via GCaMP fluorescence) to a 4 kHz auditory stimulus (Related to Figure 3).

File Name: Supplementary Movie 3
Description: Wide-area HbT or CBV response (via 570 nm IOS) to a 4 kHz auditory stimulus (Related to Figure 3).

File Name: Supplementary Movie 4
Description: Wide-area CBF response (via LSC) to a 4 kHz auditory stimulus (Related to Figure 3).

File Name: Supplementary Movie 5
Description: Wide-area dHb response (via via 680 nm IOS) to a 4 kHz auditory stimulus (Related to Figure 3). The series of Supplementary Movies 2-5 corresponds to the panel of time lapse images shown in Fig. 3d. Images in each video were normalized to 0.1% of their dynamic range for visualization purposes and displayed using a jet color-map (i.e. for each parameter, low values are in dark blue and high values in red). All playback is at 1x speed.

File Name: Supplementary Movie 6
Description: Fluorescent tracer kinetics (Related to Figure 5). Movie showing the arrival of the intravenously administered fluorescent tracer (dextran-FITC). Images were normalized to 0.1% of the dynamic range for visualization purposes and displayed using a gray-scale color-map (i.e. low intensity values are in black, while high intensity values are in white). Playback is at 1x speed.

File Name: Supplementary Movie 7
Description: Red blood cell flux in a mouse ear vessel plexus (Related to Figure 7). Movie of a fluorescent tracer (dextran-FITC) enhanced microvessel plexus from the mouse ear. One can visualize the magnitude and direction of RBC flux (dark moving spots against a bright fluorescent background) in each vessel segment using the high magnification adapter. Images were normalized to 0.1% of their dynamic range for visualization purposes.
